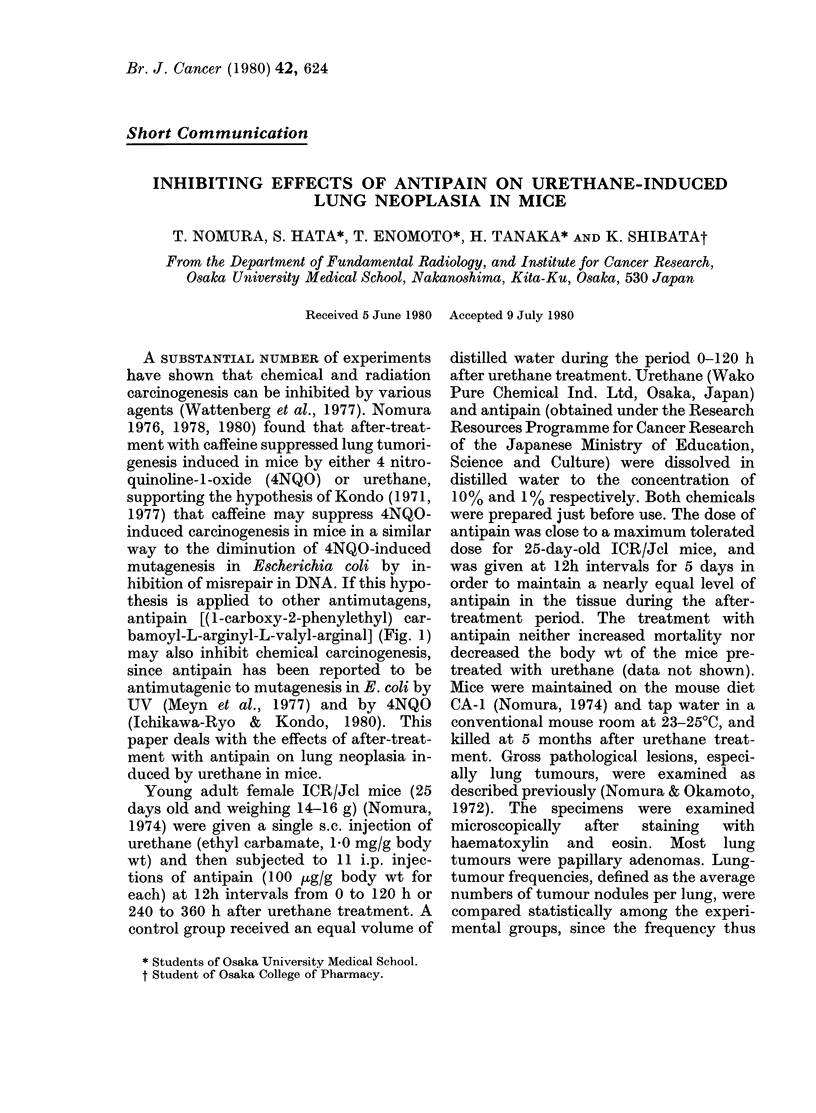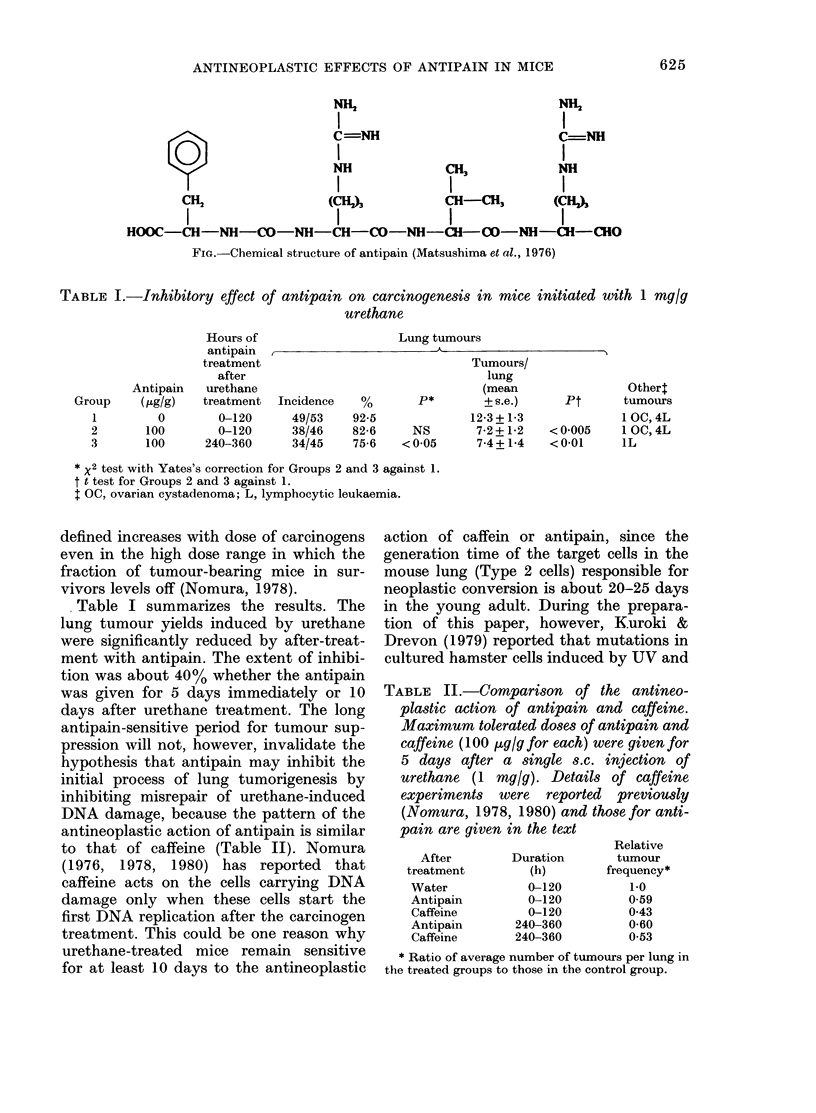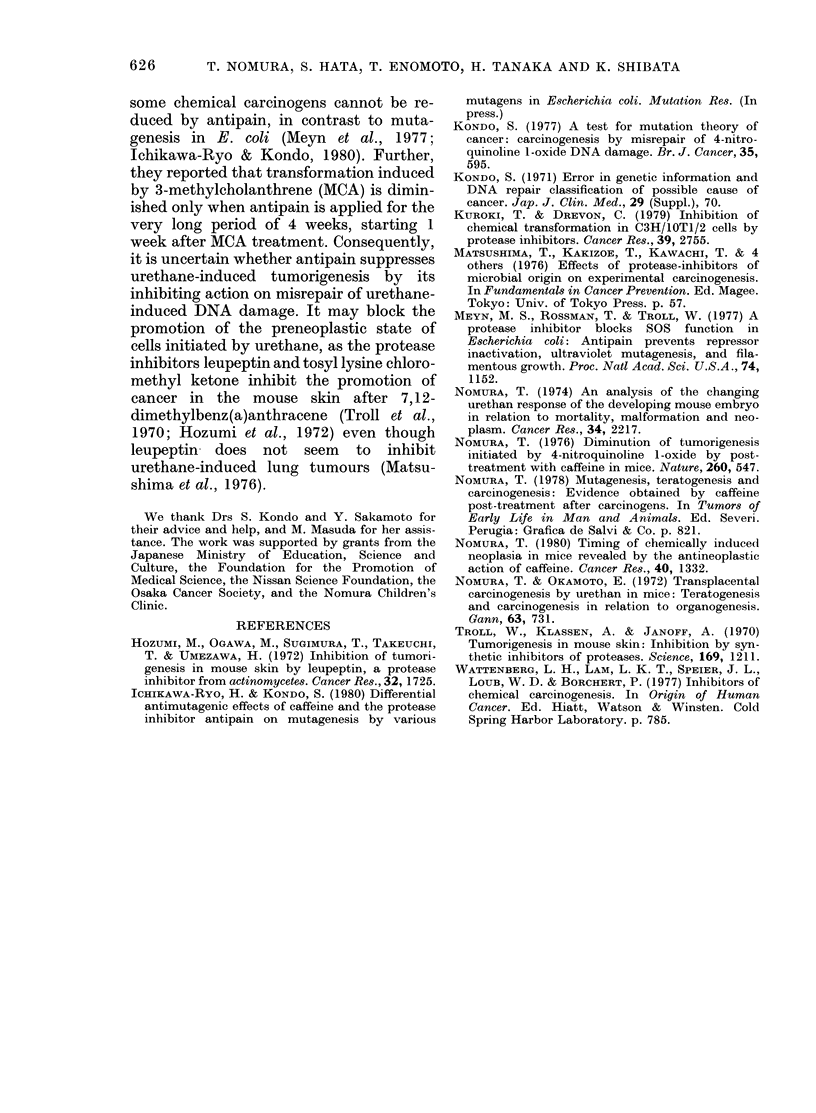# Inhibiting effects of antipain on urethane-induced lung neoplasia in mice.

**DOI:** 10.1038/bjc.1980.289

**Published:** 1980-10

**Authors:** T. Nomura, S. Hata, T. Enomoto, H. Tanaka, K. Shibata


*Br. J. Cancer* (1980) **42**, 624

***Short Communication***

### INHIBITING EFFECTS OF ANTIPAIN ON URETHANE-INDUCED LUNG NEOPLASIA IN MICE

T. NOMURA, S. HATA*, T. ENOMOTO*, H. TANAKA* AND K. SHIBATA†

*From the Department of Fundamental Radiology, and Institute for Cancer Research, Osaka University Medical School, Nakanoshima, Kita-Ku, Osaka, 530 Japan*

Received 5 June 1980 Accepted 9 July 1980

A SUBSTANTIAL NUMBER of experiments have shown that chemical and radiation carcinogenesis can be inhibited by various agents (Wattenberg *et al.*, 1977). Nomura 1976, 1978, 1980) found that after-treatment with caffeine suppressed lung tumorigenesis induced in mice by either 4 nitroquinoline-1-oxide (4NQO) or urethane, supporting the hypothesis of Kondo (1971, 1977) that caffeine may suppress 4NQO-induced carcinogenesis in mice in a similar way to the diminution of 4NQO-induced mutagenesis in *Escherichia coli* by inhibition of misrepair in DNA. If this hypothesis is applied to other antimutagens, antipain [(1-carboxy-2-phenylethyl) carbamoyl-L-arginyl-L-valyl-arginal] (Fig. 1) may also inhibit chemical carcinogenesis, since antipain has been reported to be antimutagenic to mutagenesis in *E. coli* by UV (Meyn *et al.*, 1977) and by 4NQO (Ichikawa-Ryo & Kondo, 1980). This paper deals with the effects of after-treatment with antipain on lung neoplasia induced by urethane in mice.

Young adult female ICR/Jcl mice (25 days old and weighing 14–16 g) (Nomura, 1974) were given a single s.c. injection of urethane (ethyl carbamate, 1·0 mg/g body wt) and then subjected to 11 i.p. injections of antipain (100 $\mu$g/g body wt for each) at 12h intervals from 0 to 120 h or 240 to 360 h after urethane treatment. A control group received an equal volume of distilled water during the period 0–120 h after urethane treatment. Urethane (Wako Pure Chemical Ind. Ltd, Osaka, Japan) and antipain (obtained under the Research Resources Programme for Cancer Research of the Japanese Ministry of Education, Science and Culture) were dissolved in distilled water to the concentration of 10% and 1% respectively. Both chemicals were prepared just before use. The dose of antipain was close to a maximum tolerated dose for 25-day-old ICR/Jcl mice, and was given at 12h intervals for 5 days in order to maintain a nearly equal level of antipain in the tissue during the after-treatment period. The treatment with antipain neither increased mortality nor decreased the body wt of the mice pretreated with urethane (data not shown). Mice were maintained on the mouse diet CA-1 (Nomura, 1974) and tap water in a conventional mouse room at 23–25°C, and killed at 5 months after urethane treatment. Gross pathological lesions, especially lung tumours, were examined as described previously (Nomura & Okamoto, 1972). The specimens were examined microscopically after staining with haematoxylin and eosin. Most lung tumours were papillary adenomas. Lung-tumour frequencies, defined as the average numbers of tumour nodules per lung, were compared statistically among the experimental groups, since the frequency thus

* Students of Osaka University Medical School.
† Student of Osaka College of Pharmacy.

FIG.—Chemical structure of antipain (Matsushima *et al.*, 1976)

TABLE I.—*Inhibitory effect of antipain on carcinogenesis in mice initiated with* 1 *mg/g urethane*

| | | | Lung tumours | | | | | |
|---|---|---|---|---|---|---|---|---|
| Group | Antipain (μg/g) | Hours of antipain treatment after urethane treatment | Incidence | % | $P$* | Tumours/lung (mean ± s.e.) | $P$† | Other‡ tumours |
| 1 | 0 | 0–120 | 49/53 | 92·5 | | 12·3 ± 1·3 | | 1 OC, 4L |
| 2 | 100 | 0–120 | 38/46 | 82·6 | NS | 7·2 ± 1·2 | < 0·005 | 1 OC, 4L |
| 3 | 100 | 240–360 | 34/45 | 75·6 | < 0·05 | 7·4 ± 1·4 | < 0·01 | 1L |

* $\chi^2$ test with Yates's correction for Groups 2 and 3 against 1.
† *t* test for Groups 2 and 3 against 1.
‡ OC, ovarian cystadenoma; L, lymphocytic leukaemia.

defined increases with dose of carcinogens even in the high dose range in which the fraction of tumour-bearing mice in survivors levels off (Nomura, 1978).

Table I summarizes the results. The lung tumour yields induced by urethane were significantly reduced by after-treatment with antipain. The extent of inhibition was about 40% whether the antipain was given for 5 days immediately or 10 days after urethane treatment. The long antipain-sensitive period for tumour suppression will not, however, invalidate the hypothesis that antipain may inhibit the initial process of lung tumorigenesis by inhibiting misrepair of urethane-induced DNA damage, because the pattern of the antineoplastic action of antipain is similar to that of caffeine (Table II). Nomura (1976, 1978, 1980) has reported that caffeine acts on the cells carrying DNA damage only when these cells start the first DNA replication after the carcinogen treatment. This could be one reason why urethane-treated mice remain sensitive for at least 10 days to the antineoplastic action of caffein or antipain, since the generation time of the target cells in the mouse lung (Type 2 cells) responsible for neoplastic conversion is about 20–25 days in the young adult. During the preparation of this paper, however, Kuroki & Drevon (1979) reported that mutations in cultured hamster cells induced by UV and

TABLE II.—*Comparison of the antineoplastic action of antipain and caffeine. Maximum tolerated doses of antipain and caffeine* (100 *μg/g for each*) *were given for* 5 *days after a single s.c. injection of urethane* (1 *mg/g*). *Details of caffeine experiments were reported previously* (*Nomura,* 1978, 1980) *and those for antipain are given in the text*

| After treatment | Duration (h) | Relative tumour frequency* |
|---|---|---|
| Water | 0–120 | 1·0 |
| Antipain | 0–120 | 0·59 |
| Caffeine | 0–120 | 0·43 |
| Antipain | 240–360 | 0·60 |
| Caffeine | 240–360 | 0·53 |

* Ratio of average number of tumours per lung in the treated groups to those in the control group.

some chemical carcinogens cannot be reduced by antipain, in contrast to mutagenesis in *E. coli* (Meyn *et al.*, 1977; Ichikawa-Ryo & Kondo, 1980). Further, they reported that transformation induced by 3-methylcholanthrene (MCA) is diminished only when antipain is applied for the very long period of 4 weeks, starting 1 week after MCA treatment. Consequently, it is uncertain whether antipain suppresses urethane-induced tumorigenesis by its inhibiting action on misrepair of urethane-induced DNA damage. It may block the promotion of the preneoplastic state of cells initiated by urethane, as the protease inhibitors leupeptin and tosyl lysine chloromethyl ketone inhibit the promotion of cancer in the mouse skin after 7,12-dimethylbenz(a)anthracene (Troll *et al.*, 1970; Hozumi *et al.*, 1972) even though leupeptin does not seem to inhibit urethane-induced lung tumours (Matsushima *et al.*, 1976).

We thank Drs S. Kondo and Y. Sakamoto for their advice and help, and M. Masuda for her assistance. The work was supported by grants from the Japanese Ministry of Education, Science and Culture, the Foundation for the Promotion of Medical Science, the Nissan Science Foundation, the Osaka Cancer Society, and the Nomura Children's Clinic.

### REFERENCES

HOZUMI, M., OGAWA, M., SUGIMURA, T., TAKEUCHI, T. & UMEZAWA, H. (1972) Inhibition of tumorigenesis in mouse skin by leupeptin, a protease inhibitor from *actinomycetes*. *Cancer Res.*, **32**, 1725.

ICHIKAWA-RYO, H. & KONDO, S. (1980) Differential antimutagenic effects of caffeine and the protease inhibitor antipain on mutagenesis by various mutagens in *Escherichia coli*. *Mutation Res.* (In press.)

KONDO, S. (1977) A test for mutation theory of cancer: carcinogenesis by misrepair of 4-nitroquinoline 1-oxide DNA damage. *Br. J. Cancer*, **35**, 595.

KONDO, S. (1971) Error in genetic information and DNA repair classification of possible cause of cancer. *Jap. J. Clin. Med.*, **29** (Suppl.), 70.

KUROKI, T. & DREVON, C. (1979) Inhibition of chemical transformation in C3H/10T1/2 cells by protease inhibitors. *Cancer Res.*, **39**, 2755.

MATSUSHIMA, T., KAKIZOE, T., KAWACHI, T. & 4 others (1976) Effects of protease-inhibitors of microbial origin on experimental carcinogenesis. In *Fundamentals in Cancer Prevention*. Ed. Magee. Tokyo: Univ. of Tokyo Press. p. 57.

MEYN, M. S., ROSSMAN, T. & TROLL, W. (1977) A protease inhibitor blocks SOS function in *Escherichia coli*: Antipain prevents repressor inactivation, ultraviolet mutagenesis, and filamentous growth. *Proc. Natl Acad. Sci. U.S.A.*, **74**, 1152.

NOMURA, T. (1974) An analysis of the changing urethan response of the developing mouse embryo in relation to mortality, malformation and neoplasm. *Cancer Res.*, **34**, 2217.

NOMURA, T. (1976) Diminution of tumorigenesis initiated by 4-nitroquinoline 1-oxide by post-treatment with caffeine in mice. *Nature*, **260**, 547.

NOMURA, T. (1978) Mutagenesis, teratogenesis and carcinogenesis: Evidence obtained by caffeine post-treatment after carcinogens. In *Tumors of Early Life in Man and Animals*. Ed. Severi. Perugia: Grafica de Salvi & Co. p. 821.

NOMURA, T. (1980) Timing of chemically induced neoplasia in mice revealed by the antineoplastic action of caffeine. *Cancer Res.*, **40**, 1332.

NOMURA, T. & OKAMOTO, E. (1972) Transplacental carcinogenesis by urethan in mice: Teratogenesis and carcinogenesis in relation to organogenesis. *Gann*, **63**, 731.

TROLL, W., KLASSEN, A. & JANOFF, A. (1970) Tumorigenesis in mouse skin: Inhibition by synthetic inhibitors of proteases. *Science*, **169**, 1211.

WATTENBERG, L. H., LAM, L. K. T., SPEIER, J. L., LOUB, W. D. & BORCHERT, P. (1977) Inhibitors of chemical carcinogenesis. In *Origin of Human Cancer*. Ed. Hiatt, Watson & Winsten. Cold Spring Harbor Laboratory. p. 785.